# QTL and candidate genes associated with leaf anion concentrations in response to phosphate supply in *Arabidopsis thaliana*

**DOI:** 10.1186/s12870-019-1996-3

**Published:** 2019-09-18

**Authors:** Mohamed El-Soda, Charles Neris Moreira, Nakai Goredema-Matongera, Diaan Jamar, Maarten Koornneef, Mark G. M. Aarts

**Affiliations:** 1 Department of Genetics, Faculty of Agriculture, Cairo University, Giza, 12613 Egypt; 2 Laboratory of Genetics, Wageningen University and Research, Droevendaalsesteeg 1, 6708 PB Wageningen, The Netherlands; 3 Department of Research and Specialist Services, Maize Breeding Programme, Crop Breeding Institute, P. O. Box CY550 Causeway, Harare, Zimbabwe; 4 Laboratory of Plant Physiology, Wageningen University and Research, Droevendaalsesteeg 1, 6708 PB Wageningen, The Netherlands

**Keywords:** Anion concentration, Phosphate deficiency, QTL and association mapping, QTL x E

## Abstract

**Background:**

Phosphorus is often present naturally in the soil as inorganic phosphate, Pi, which bio-availability is limited in many ecosystems due to low soil solubility and mobility. Plants respond to low Pi with a Pi Starvation Response, involving Pi sensing and long-distance signalling. There is extensive cross-talk between Pi homeostasis mechanisms and the homeostasis mechanism for other anions in response to Pi availability.

**Results:**

Recombinant Inbred Line (RIL) and Genome Wide Association (GWA) mapping populations, derived from or composed of natural accessions of *Arabidopsis thaliana*, were grown under sufficient and deficient Pi supply. Significant treatment effects were found for all traits and significant genotype x treatment interactions for the leaf Pi and sulphate concentrations. Using the RIL/QTL population, we identified 24 QTLs for leaf concentrations of Pi and other anions, including a major QTL for leaf sulphate concentration (*SUL2*) mapped to the bottom of chromosome (Chr) 1. GWA mapping found 188 SNPs to be associated with the measured traits, corresponding to 152 genes. One of these SNPs, associated with leaf Pi concentration, mapped to *PP2A-1*, a gene encoding an isoform of the catalytic subunit of a protein phosphatase 2A. Of two additional SNPs, associated with phosphate use efficiency (PUE), one mapped to AT5G49780, encoding a leucine-rich repeat protein kinase involved in signal transduction, and the other to *SIZ1*, a gene encoding a SUMO E3 ligase, and a known regulator of P starvation-dependent responses. One SNP associated with leaf sulphate concentration was found in *SULTR2;1,* encoding a sulphate transporter, known to enhance sulphate translocation from root to shoot under P deficiency. Finally, one SNP was mapped to *FMO GS-OX4*, a gene encoding glucosinolate S-oxygenase involved in glucosinolate biosynthesis, which located within the confidence interval of the *SUL2* locus.

**Conclusion:**

We identified several candidate genes with known functions related to anion homeostasis in response to Pi availability. Further molecular studies are needed to confirm and validate these candidate genes and understand their roles in examined traits. Such knowledge will contribute to future breeding for improved crop PUE .

**Electronic supplementary material:**

The online version of this article (10.1186/s12870-019-1996-3) contains supplementary material, which is available to authorized users.

## Background

Present intensive field crop cultivation practices lead to land degradation, lowering soil fertility and productivity, while depending heavily on the extensive use of fertilizers. To meet food demands for the increasing world population, future agriculture may need to expand to currently uncultivated marginal lands. Alternatively, more resource efficient cultivation methods and corresponding crop varieties are to be developed. In either case, the crops of the future will need to deal with a lower input of macronutrients, such phosphorus (P), nitrogen, potassium and sulphur, which are essential for plant growth, development and productivity [1, 2]. A more efficient uptake and use of nutrients by crops will be an important target for future plant breeding in order to develop novel, sustainable, crop varieties.

P in soil is largely immobile, with inorganic phosphate (Pi) in the soil solution occurring as a very small fraction in total soil P [3]. Only Pi can be taken up by plants and microorganisms and is indispensable for a thriving ecosystem, providing nutrition for the most important biological processes, such as photosynthesis, energy storage, carbon fixation, lipid metabolism and respiration [4, 5]. During millions of years of evolution, plants have developed elegant nutrient acquisition strategies to efficiently acquire and use Pi under P-limited soil conditions [6, 7]. Still, Pi is taken up by roots at a relatively low efficiency due to its low solubility and mobility in soils [8–10], making Pi availability one of the most limiting factors for plant growth and productivity worldwide. One of the reasons is soil pH, as Pi is mainly available within 6.5 < pH < 7.5. In acidic soils, Fe/Al-P-minerals are more common and form insoluble compounds which are poorly absorbed by plants [11]. In more alkaline soils, Ca/Mg-P-minerals are mostly precipitated, making Pi unavailable [10]. Despite the excessive amounts of Pi fertilizers currently applied, on average only 10–20% of applied Pi may be used by crops, while the remainder will be lost by leaching into the groundwater or by long-term immobilization in soil, both leading to substantial socioeconomic and environmental costs [12].

To overcome P deficiency, plants can produce and release organic acids from roots, which can solubilize Pi [13]. Several reports have shown the release of citrate, malate and oxalate from roots of cowpea [14], white lupin [15, 16], and soybean [17] upon low Pi supply. Overall, the P-deficiency-induced changes in organic acid metabolism differed between roots and leaves [18], however, it is not well established how P deficiency affects the accumulation of organic acids in the shoot and their release by roots. It is also interesting to determine the concentrations of phytate (IP6), nitrate, and sulphate, as these compounds may be affected by Pi supply and may be regulated by similar genes involved in control of low Pi response. For example, a major quantitative trait locus (QTL) for both IP6 and Pi concentrations in seeds and in leaves was previously detected in *Arabidopsis thaliana* (Arabidopsis) [19]. Although organic acids affect Pi uptake in roots it is not unrealistic to assume that genetic variation for their production is also expressed and functional in leaves. At least for sulphate and Pi flux control a common regulatory step was described [20]. Furthermore, cross talk between sensing of Pi and nitrate status has been reported recently [21].

There is an increasing demand to develop new crop varieties that are more efficient in the uptake, transport, storage, mobilization and/or use of Pi [22]. Plants store approximately 90% of their P in seeds, mostly in the form of IP6, and approximately 10% in their leaves. During germination, seeds express the enzymes to degrade IP6 and release Pi again. IP6 in food or feed is poorly digested by humans or non-ruminants, leading to additional losses of P in the food chain and increasing environmental pollution. Thus, there is also a need for crops with a reduced accumulation of IP6 in seeds, and which instead store P in more digestible forms [8, 19, 23].

Clues on which genes contribute to more Pi efficient plants may be found by examining natural genetic diversity for enhanced Pi uptake and Pi use efficiency in model as well as wild plant species or ancient crop germplasm [2, 24]. High Pi use efficiency plants can increase productivity and lead to good performance on low Pi soils. However, good performance in low Pi conditions and Pi use efficiency are complex traits, which are affected by many factors that seem to be either directly or indirectly connected by plant responses to Pi limitation [25, 26]. Plants have evolved a highly efficient Pi starvation response, which involves Pi sensing and long distance Pi signalling, to promote Pi use efficiency [26–28]. Several of the factors involved in sensing Pi deficiency, and responding to it, are already known (as has recently been reviewed in detail by [26]). It involves several transcription factors and miRNAs, controlling transcriptional responses of genes involved in Pi acquisition, remobilization, distribution and (re)sequestration of Pi in the plant once sufficient Pi has been acquired. Excess Pi is stored in the vacuole from where it can be remobilized to the cytoplasm in case of additional deficiencies [20, 27, 29].

The ability of a genotype to adapt to the environment by producing distinct morpho-physiological and biochemical phenotypes in different environments depends on the developmental stage and is known as phenotypic plasticity. Natural variation in the phenotypic plasticity is known as genotype by environment interaction (GxE). Evaluating Arabidopsis responses to different levels of Pi availability, as the environmental factor, provides a solid foundation for the genetic improvement of stable Arabidopsis productivity and helps to identify superior alleles across different Pi levels [30]. Several approaches have been taken to understand Pi homeostasis including those focused on resolving regulatory networks [31–34]. In addition, novel factors involving Pi homeostasis can also be unravelled by dissecting the genetic variation found within the germplasm of the species, either by examining the progeny of biparental crosses, e.g. Recombinant Inbred Line (RIL) populations, in classical linkage mapping studies [35], or across a larger pool of genotypes by Genome-Wide Association Studies (GWAS) [36]. Such approaches will identify QTLs and eventually the underlying causal allelic variation contributing to the phenotypic variation. However, although GWAS allow high accuracy mapping of the underlying loci, when compared with RIL/QTL analysis, it often lacks the power to detect the effect of rare alleles, even if they have large phenotypic effects, as well as alleles which are confounded by population structure [37–39]. Therefore, the combination of both GWAS and RIL/QTL mapping to analyse a trait will give high accuracy mapping and combine the advantages of both approaches by accounting for false positives and avoiding false negatives [30, 39]. Incorporating environmental factors, in this case Pi availability, in RIL and GWA mapping models allows the mapping of QTLs and their interaction with the environment (QTL × E) [30]. This in turn helps distinguishing a QTL with synergistic pleiotropic effects, i.e. a QTL with positive effects of one allele on two or more traits, from a QTL with antagonistic pleiotropic effects, i.e., a QTL with opposite effects of both alleles on two or more traits in which one allele enhances one trait and the other allele enhances other traits. In addition, it facilitates the mapping of a conditional neutrality QTL, i.e. a QTL showing an effect on a trait in one environment, but without effect in other environments [30]. Understanding the effects of each QTL is crucial when selecting for desirable QTLs during marker assisted breeding programs.

Here, we studied the genetics of leaf production (measured as dry weight; DW) of different Arabidopsis genotypes, as well as their Pi concentration and phosphate use efficiency (PUE) of plants grown under Pi sufficient (+Pi) and Pi deficient (−Pi) treatments. In addition we studied the concentration of phytate, nitrate, citrate, oxalate and sulphate in the same plant parts. We studied these traits to identify QTLs, and candidate genes that may underlie these QTLs, using traditional linkage mapping in a RIL population (RIL/QTL) and GWAS analysis of a HapMap diversity panel (HapMap/GWAS). We also examined the G x E interaction effects of these loci in the two treatments.

## Results

### Phenotyping the mapping populations

Upon growing the F6 RIL/QTL population, composed of 164 lines and derived from crossing the Shahdara (Sha) and Columbia (Col) accessions [76], under both sufficient (+Pi) and deficient phosphate (−Pi) supply, significant effects of the -Pi treatment were observed for all traits as well as significant genotype x treatment interactions for the leaf Pi, phytate and sulphate concentrations. Upon growing the 360 diverse accessions of the HapMap/GWAS population, which include the Sha and Col accessions, [77] subjected to similar treatments, again significant treatment effects and genotype x treatment interactions were found for all traits (Table 1 and Additional file 1). The heritabilities of these traits ranged between 0.42–0.73, in case of the RIL/QTL population, and between 0.38–0.80 for the HapMap/GWAS population (Table 1).

**Table 1:** The performance of the parental lines and RIL/QTL (a) and GWAS/HapMap (b) populations under phosphate sufficient (+Pi) and phosphate deficient (−Pi) treatments (T)

(a)											
		Parents		RIL/QTL population							
	T	Sha	Col	Min	Max	Mean	Std	H^2^	ANOVA		
									G	T	GxT
DW (g/plant)	+Pi	0.032	0.051	0.007	0.094	0.037	0.015	0.50	0.00	0.02	0.54
	-Pi	0.024	0.042	0.009	0.070	0.039	0.012	0.36			
PHO (mg/g DW)	+ Pi	13.8	17.9	8.14	35. 5	20.6	3.7	0.41	0.00	0.00	0.00
	- Pi	3.39	4.93	2.10	10.49	4.37	1.29	0.33			
PUE	+ Pi	0.010	0.013	0.004	0.099	0.030	0.02	–	–	–	–
	- Pi	0.421	0.321	0.233	1.643	0.901	0.27	–			
SUL (mg/g DW)	+ Pi	9.2	16.4	4.4	44.9	14.7	6.0	0.77	0.00	0.00	0.05
	- Pi	12.8	14.1	3.3	33.1	11.5	5.3	0.44			
(b)											
		HapMap/GWAS population									
	T	Sha	Col	Min	Max	Mean	Std	H^2^	ANOVA		
									G	T	GxT
DW (g/plant)	+Pi	0.038	0.047	0.011	0.076	0.039	0.01	0.65	0.00	0.00	0.00
	-Pi	0.019	0.023	0.005	0.044	0.022	0.01	0.48			
PHO (mg/g DW)	+ Pi	13.0	13.5	5.15	24.9	13.1	3.2	0.37	0.00	0.00	0.00
	- Pi	1.49	3.39	0.67	10.3	2.92	1.14	0.22			
PUE	+ Pi	0.077	0.075	0.040	0.194	0.079	0.02	–	–	–	–
	- Pi	0.671	0.295	0.097	0.923	0.387	0.14	–			
SUL (mg/g DW)	+ Pi	10.4	12.1	3.2	27.3	11.7	3.6	0.41	0.00	0.00	0.00
	- Pi	14.6	19.5	2.3	39.8	11.2	4.7	0.38			

DW = total rosette dry weight, PHO = leaf Pi concentration, PUE = phosphate use efficiency determined as shoot dry weight / PHO, and SUL = leaf sulphate concentration. Min and Max indicate the lowest and highest values; mean and standard deviation (Std) is indicated for all lines; H^2^ indicates broad sense heritability. In the ANOVA table, G, T, and GxT, refer to respectively genotype, treatment and genotype x treatment interaction and each of them is considered significant when the *P* value is ≤0.05

**Fig. 1:**
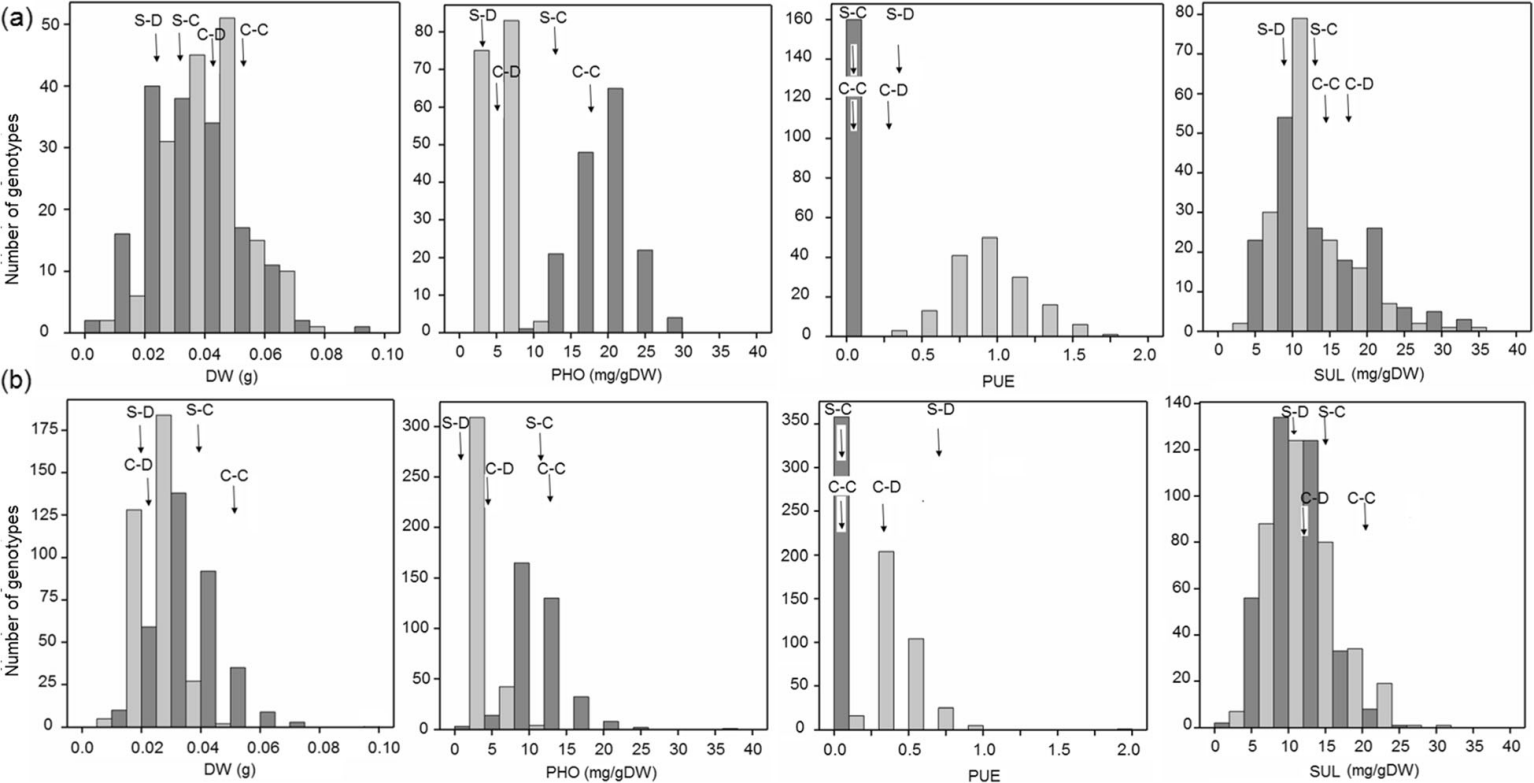
Frequency distributions of the non-normalized values of the traits measured for the RIL/QTL (**a**) and HapMap/GWAS (**b**) populations. Sha-C and Col-C refer to the values of the parental lines grown with sufficient Pi and Sha-D and Col-D to the values of the parental lines grown with deficient Pi, all indicated with arrows. Data for the sufficient Pi treatment are presented with dark grey bars and data from the deficient Pi treatment with light grey bars. The vertical axes indicate the numbers of genotypes per trait value class and the horizontal axes indicate the different trait value classes. DW = total rosette dry weight, PHO = leaf Pi concentration, PUE = phosphate use efficiency determined as shoot dry weight / PHO, and SUL = leaf sulphate concentration

Frequency distributions of the measured traits for both populations showed transgression beyond both parental lines for all traits (Fig. 1 and Additional file 2). Within each population there was a positive correlation when comparing each trait measured in the +Pi treatment with the trait measured in the –Pi treatment (Table 2 and Additional file 1). In both treatments and in both populations, the leaf Pi and IP6 concentrations were also positively correlated. Similarly, the leaf nitrate, sulphate and citrate concentrations were positively correlated with each other and negatively correlated with leaf oxalate concentrations in both populations in both treatments.

**Table 2:** Pearson correlations for the analysed traits in the RIL/QTL (a) and the HapMap/GWAS (b) populations measured under phosphate sufficient (+Pi) and phosphate deficient (−Pi) treatments

		(a) RIL/QTL							(b) GWAS/HapMap						
		+Pi				-Pi			+Pi				-Pi		
	Trait	DW	PHO	PUE	SUL	DW	PHO	PUE	DW	PHO	PUE	SUL	DW	PHO	PUE
+Pi	DW	1							1						
	PHO	0.01	1						−0.18^**^	1					
	PUE	0.92^**^	−0.35^**^	1					0.59^**^	−0.65^**^	1				
	SUL	−0.14	0.03	−0.13	1				− 0.16^**^	0.35^**^	−0.30^**^	1			
-Pi	DW	0.49^**^	0.01	.0.48^**^	−0.25^**^	1			0.56^**^	− 0.08	0.34^**^	−0.08	1		
	PHO	−0.27^**^	0.04	−0.28^**^	0.11	−0.28^**^	1		−0.05	0.11^*^	−0.07	−0.04	0.04	1	
	PUE	0.51^**^	−0.06	0.54^**^	−0.20^*^	0.81^**^	−0.64^**^	1	0.35^**^	−0.13^*^	0.23^**^	−0.07	0.52^**^	−0.68^**^	1
	SUL	−0.23^**^	−0.05	− 0.19^*^	0.60^**^	− 0.39^**^	0.48^**^	− 0.42^**^	−0.06	0.07	−0.07	0.18^**^	0.02	0.28^**^	−0.22^**^

DW = total rosette dry weight, PHO = leaf Pi concentration, PUE = phosphate use efficiency determined as shoot dry weight / PHO, and SUL = leaf sulphate concentration. Correlations that are significant at *p* < 0.05 and *p* < 0.01 levels are indicated with * and **, respectively

### QTL mapping in the RIL/QTL population

**Fig. 2:**
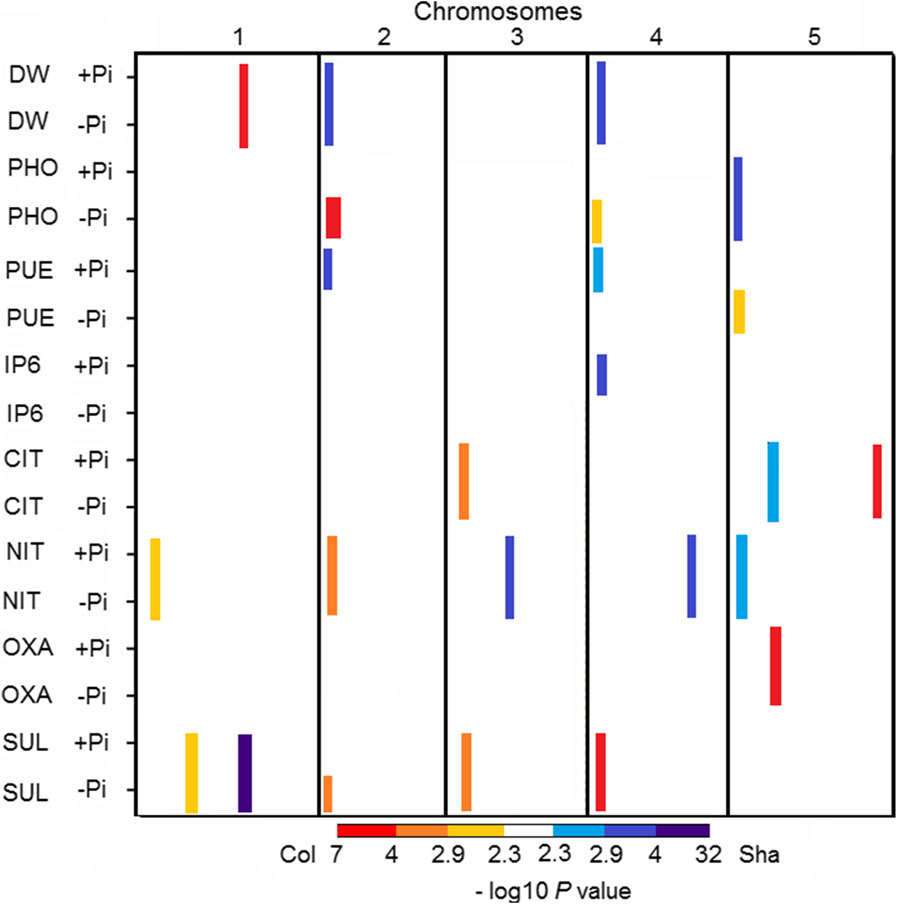
A heat-map showing the –log_10_(*P*) profiles of the measured traits in the RIL/QTL population. Columns indicate the five Arabidopsis chromosomes in centiMorgans, ascending from left to right; rows indicate individual trait –log_10_(*P*) profiles. A colour scale is used to indicate the QTL significance corresponding to the –log_10_(*P*) score: gold, orange, and red represent a positive effect on the trait value from the Col allele; light, medium and dark blue represent a positive effect on the trait value from the Sha allele. +Pi and -Pi refer to phosphate sufficient and phosphate deficient treatments, respectively. DW = total rosette dry weight, PHO = leaf Pi concentration, PUE = phosphate use efficiency, IP6 = leaf phytate concentration, CIT = leaf citrate concentration, NIT = leaf nitrate concentration, and SUL = leaf sulphate concentration

In total, 24 significant QTLs were mapped for the measured traits using the RIL/QTL population, of which 9 QTLs showed significant QTLxE effects (Fig. 2, Table 3 and Additional file 1). Three QTL clusters were mapped to the top of Chr 2, 4 and 5 respectively. The QTLs for leaf Pi concentration, *PHO1*, *PHO2* and *PHO3*, co-located with QTLs for phosphate use efficiency, *PUE1*, *PUE2* and *PUE3*, respectively. On the top of Chr 2, possibly antagonistic pleiotropic effects were observed between *PHO1* and QTLs for leaf nitrate concentration *NIT2* and leaf sulphate concentration *SUL3*, with positive effects from the Col allele, and *DW2* and *PUE1*, with positive effects from the Sha allele. One major QTL, *SUL2*, was mapped to the bottom of Chr 1 with a significant QTLxE interaction explaining 42.9 and 33.7% of the variation in leaf sulphate concentration under +Pi and –Pi treatments, respectively. The Sha allele of *SUL2* confers a higher leaf sulphate concentration than the Col allele in both Pi treatments. *SUL2* co-localised with *DW1,* a QTL with the Col allele conferring higher DW, indicating possibly antagonistic pleiotropic effects. Seven QTLs showed conditional neutrality, such as *PHO1*, *PHO2*, *PUE3*, and *SUL3*, with positive effects from the Col allele, and *PUE1*, *PUE2*, and *IP6.1*, with positive effects from the Sha allele. *IP6.1* co-located with *SUL5* on the top of Chr 4 with opposite allelic effects in the +P treatment.

**Table 3:** QTLs detected in the RIL/QTL population for the measured traits under +Pi and –Pi treatments

Trait	QTLs						+ Pi		- Pi	
	Name	Chr	Marker	Position in cM	–log_10_(*P*)	QTLxE	R^2^	Effect	R^2^	Effect
DW (g/plant)	*DW1*	1	c1_23381	73.2	3.8	no	3.5	0.003	7.1	0.003
	*DW2*	2	c2_00593	0	4.8	no	4.7	−0.003	9.4	−0.003
	*DW3*	4	c4_05850	22.2	3.5	no	3.2	−0.003	6.4	−0.003
PHO (mg/g DW)	*PHO1*	2	c2_02365	7.2	4.1	yes	–	–	8.5	0.38
	*PHO2*	4	c4_00641	7	2.6	yes	–	–	4.9	0.29
	*PHO3*	5	c5_02900	9	3.3	yes	6.7	−1.23	2.6	−0.21
PUE	*PUE1*	2	c2_02365	7.2	3.8	yes	8.3	−0.006	–	–
	*PUE2*	4	c4_00641	7	2.3	yes	4.5	−0.004	–	–
	*PUE3*	5	c5_00576	0	2.6	yes	–	–	2.2	0.067
SUL (mg/g DW)	*SUL1*	1	C1P38	37.6	2.7	no	2.3	1.04	4.3	1.04
	*SUL2*	1	c1_23381	73.2	32.3	yes	42.9	−4.50	33.7	−2.91
	*SUL3*	2	c2_00593	0.0	2.9	yes	–	–	4.7	1.09
	*SUL4*	3	c3_02968	5.9	3.7	no	2.4	1.06	4.4	1.06
	*SUL5*	4	c4_04877	15.9	6.6	no	4.4	1.45	8.3	1.45

Phosphate sufficient (+Pi) and phosphate deficient (−Pi) treatments. DW = rosette dry weight, PHO = leaf Pi concentration, PUE = phosphate use efficiency, and SUL = leaf sulphate concentration. –log_10_(*P*) indicates the significance level, a threshold of –log_10_(*P*) = 2.9 is used for identification of significant QTLs, QTLxE indicates the presence or absence of QTL by environment interaction, R^2^ is the percentage of total phenotypic variance explained by each QTL. Effects with positive values represent a positive contribution of the Col allele to the trait value and those with negative values represent a positive contribution of the Sha allele to the trait value

### Mapping SNPs for anion accumulation in leaves using the HapMap/GWAS population

**Fig. 3:**
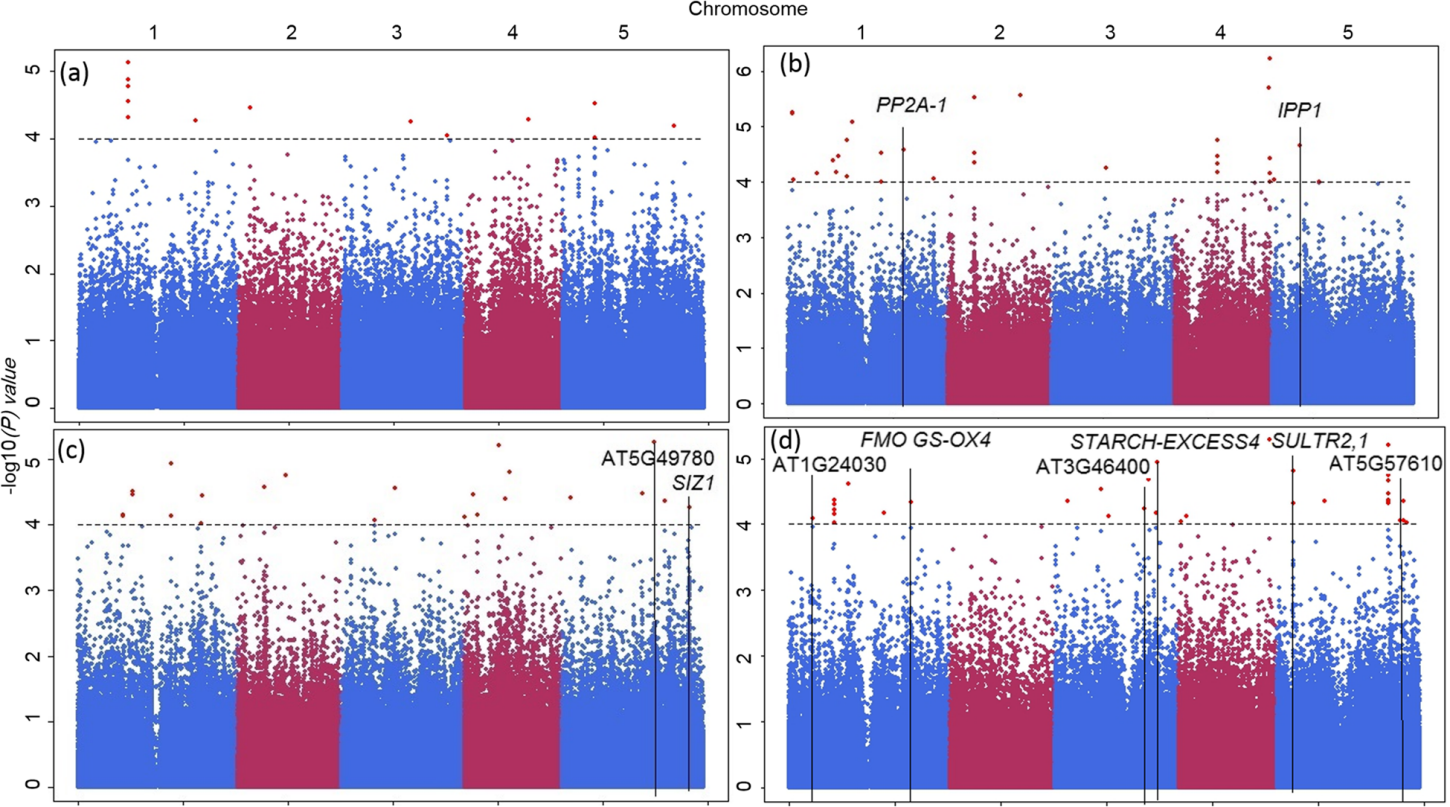
HapMap/GWA mapping using the multi-trait mixed model (MTMM) approach showing the −log_10_(*P*) values (Y-axis), for all SNPs (X-axis) The measured traits are rosette dry weight (**a**), and leaf Pi concentrations (**b**), Pi use efficiency (**c**), and leaf sulphate concentration (**d**). SNPs associated with candidate genes listed in Table 4 are indicated with vertical black lines. In each panel, the SNPs corresponding to the five Arabidopsis chromosomes are indicated in alternating blue/purple colours, with the horizontal axes indicating genome sequence positions. The −log_10_(*P*) arbitrary significance threshold of 4 is indicated with a horizontal dashed line

We identified 188 significant single nucleotide polymorphism (SNPs) with -log_10_(*P*) ≥ 4, corresponding to 152 genes (Fig. 3, Additional files 3 and 4). Table 4 presents the selected significant SNPs and their corresponding genes with known biological function, if somehow related to phosphate deficiency, as described in TAIR. For example, a significant SNP associated with Pi concentration mapped in *PROTEIN PHOSPHATASE 2A-1 (PP2A-1 -* AT1G59830) [40], a gene that encodes one of the isoforms of the catalytic subunit of protein phosphatase 2A. The non-Col allele of this SNP confers a 10 times larger phenotypic effect in the +Pi treatment than in the –Pi treatment. An additional SNP, associated with PUE, was mapped to AT5G49780 [41], which encodes a leucine-rich repeat receptor-like protein kinase. One more SNP associated with PUE was mapped to *SIZ1* (AT5G60410) [42], which encodes for a SUMO E3 ligase involved in protein degradation. This is a known regulator of P starvation-dependent responses [43]. For the two SNPs associated with PUE, the non-Col allele confers approximately a two times larger phenotypic effect than the Col allele, in both phosphate treatments.

**Table 4:** Selected candidate genes mapped in HapMap/GWAS population for anion concentration under phosphate sufficient (+Pi) and deficient (−Pi) treatments

Trait	Chr	Gene	position in kp	MAF	-log_10_(P)	β deficient	β control	Description TAIR v10	Genes in LD
PHO	1	AT1G59830	22,022	0.3	4.6	−0.11	−1.16	*PROTEIN PHOSPHATASE 2A-1 (PP2A-1).* Encodes one of the isoforms of the catalytic subunit of protein phosphatase 2A.	
	5	AT5G16440	5370	0.08	4.7	0.53	1.78	*ISOPENTENYL DIPHOSPHATE ISOMERASE 1 (IPP1*). Encodes a protein with isopentenyl diphosphate:dimethylallyl diphosphate isomerase activity.	AT5G16430
PUE	5	AT5G49780	20,232	0.05	5.3	1.06	−2.12	Leucine-rich repeat protein kinase family protein involved in protein phosphorylation.	AT5G49810 - AT5G49820 - AT5G49830
	5	AT5G60410	24,293	0.08	4.3	−0.76	−1.63	*SIZ1*, encodes a plant small ubiquitin-like modifier (SUMO) E3 ligase that is a focal controller of Pi starvation-dependent responses.	AT5G60440 - AT5G60450
SUL	1 (2)	AT1G24030	8503	0.13	4.9	−0.25	−1.50	Protein kinase superfamily protein; functions in: protein serine/threonine kinase activity, protein kinase activity, kinase activity, ATP binding; involved in: protein amino acid phosphorylation.	AT1G24040 - AT1G24060
	1	AT1G62570	23,171	0.12	4.4	−0.71	0.08	*FLAVIN-MONOOXYGENASE GLUCOSINOLATE S-OXYGENASE 4, FMO GS-OX4*. Encodes a glucosinolate S-oxygenase that catalyzes the conversion of methylthioalkyl glucosinolates to methylsulfinylalkyl glucosinolates	
	3	AT3G46400	17,073	0.14	4.2	0.64	0.67	Leucine-rich repeat protein kinase family protein; functions in: kinase activity; involved in: protein amino acid phosphorylation.	AT3G46382
	3	AT3G52180	19,351	0.46	4.9	−0.47	0.41	*STARCH-EXCESS4* Encodes a plant-specific glucan phosphatase that contains a noncatalytic carbohydrate-binding module as well as a dual specificity protein phosphatase domain	AT3G52140 - AT3G52170
	5	AT5G10180	3194	0.32	4.8	−0.56	0.10	*ARABIDOPSIS SULFATE TRANSPORTER 68, AST68, SULFATE TRANSPORTER 2;1, SULTR2;1.* Encodes a low-affinity sulfate transporter expressed in the root cap and central cylinder, where it is induced by sulfur starvation. Expression in the shoot vascular system is not induced by sulfur starvation.	AT5G10170 - AT5G10240
	5	AT5G57610	23,322	0.17	4.1	0.39	−1.14	Protein kinase superfamily protein. Involved in protein amino acid phosphorylation.	AT5G57580

PHO = leaf Pi concentration, PUE = Pi use efficiency,, SUL = leaf sulphate concentration. MAF is the minor allele frequency. -log_10_(P) indicates the significance level of association. β indicates the phenotypic effect of a SNP in (+Pi) or (−Pi) with positive values indicating a positive effect on the trait value from the Col allele. Chromosome numbers are indicated (Chr.). Numbers between brackets refer to the number of significant SNPs, the SNP positions on each chromosome are given, in kilo base pairs (kb). Both SNP position and description is based on TAIR v.10 (www.arabidopsis.org). Genes found to be in linkage disequilibrium (LD), (LD > 0.3) or within 10 kb on both sides of the significant SNP if no LD is found, are listed

An association was found between the leaf sulphate concentration and a SNP in the *SULFATE TRANSPORTER 2;1* (*SULTR2;1* - AT5G10180) gene, which is involved in sulphate uptake [44, 45]. For this locus, the effect of the non-Col allele in the –Pi treatment was approximately five times higher than in the +Pi treatment and in the opposite direction. Another association was found between a SNP mapped in the *DUAL-SPECIFICITY PROTEIN PHOSPHATASE 4* (*DSP4* - AT3G52180) gene, encoding a plant-specific glucan phosphatase, which is involved in glucosinolate biosynthesis, in protein and starch dephosphorylation [46–49], and in sulphate concentration. This SNP showed an antagonistic effect in response to phosphate availability, where Col contained the allele increasing the leaf sulphate concentration in the +Pi treatment and the non-Col allele increasing the leaf sulphate concentration in the –Pi treatment. Three genes associated with leaf sulphate concentration, AT5G57610 [50], AT3G46400 and AT1G24030 [51], encode for protein kinases involved in protein phosphorylation. For AT5G57610, the effect of the non-Col allele was approximately three times higher in the +Pi treatment than the effect of the Col allele in the –Pi treatment. The non-Col allele of AT1G24030 had a six times higher effect in the +Pi than in the –Pi treatment. One additional SNP, associated with leaf sulphate concentration, mapped at the *Relative of Early Flowering 6* gene (*REF6* - AT3G48430), which encodes a histone H3 lysine 27 demethylase [52] that acts as a positive regulator of flowering in an FLC-dependent manner [53].

### The *SUL2* QTL mapped in the RIL/QTL population is also found in the HapMap/GWAS population

**Fig. 4:**
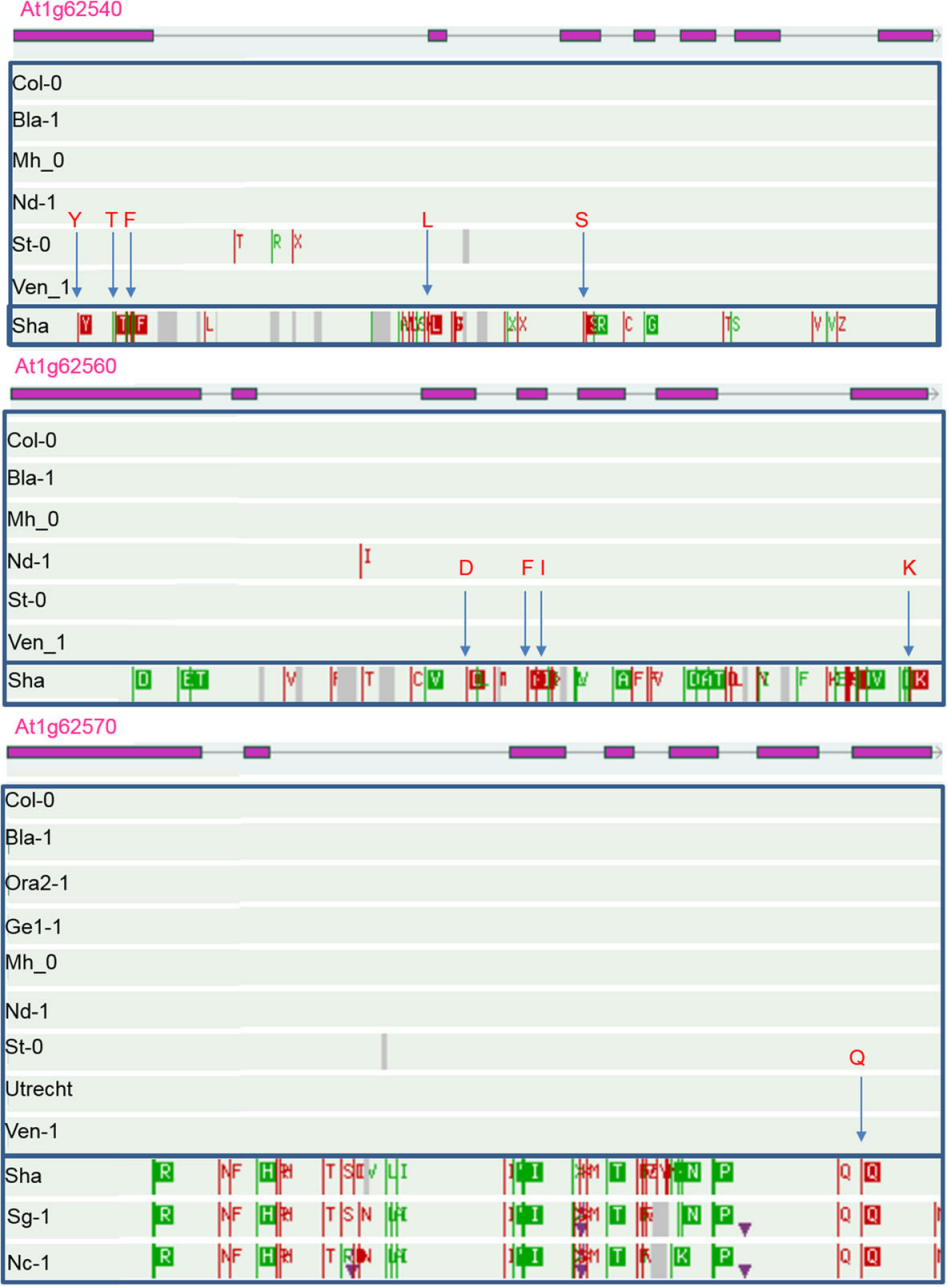
Comparison of the amino acid sequences for the three genes AT1G62540 (*FMO GS-OX2*)*,* AT1G62560 (*FMO GS-OX3*), and AT1G62570, (*FMO GS-OX4*). Comparison of the amino acid sequences for the three genes between the Col and Sha haplotypes. For each gene, exons are indicated with purple boxes and introns with lines connecting them. Amino acids differing from the Col-0 reference genome are marked in green and red. Blue arrows indicate non-synonymous amino acid differences between Col and Sha. Letters above the arrows refer to the amino acids as follows, Y = tyrosine, T = threonine, F = phenylalanine, L = leucine, S = serine, D = aspartic acid, I = isoleucine, K = lysine, Q = glutamine (picture from http://signal.salk.edu/atg1001/3.0/gebrowser.php)

HapMap/GWAS allowed a comparison to the QTLs identified in the Sha x Col RIL/QTL population, to see if any are present in both populations. This was the case for the *SUL2* locus (Table 3), for which the SNP with the highest –log_10_(*P*) value was mapped at 23,381 kilobasepair (kb). This co-located with a significant SNP at 23,171 kb (−log_10_(*P*) = 4.4) detected in the HapMap/GWAS with significant interaction with phosphate availability (Table 4). This SNP mapped to the *FLAVIN-MONOOXYGENASE GLUCOSINOLATE S-OXYGENASE 4* (*FMO GS-OX4*; AT1G62570) gene [54]. Glucosinolate S-oxygenase catalyzes the conversion of methylthioalkyl glucosinolates to methylsulfinylalkyl glucosinolates. The non-Col allele of this SNP increased leaf sulphate concentration in the -Pi treatment. No SNPs were found to be in linkage disequilibrium (LD) with *FMO GS-OX4*, however, the two neighbouring genes are from the same family, i.e. *FMO GS-OX2* and *FMO GS-OX3,* all in the confidence interval of *SUL2*. Although there are additional SNPs in these genes, five in AT1G62540 (*FMO GS-OX2*) [55] and four in AT1G62560 (*FMO GS-OX3*) [54] that are not in LD (Fig. 4), these are not significantly associated with leaf sulphate concentration, and are unlikely to contribute to the associated phenotypic difference. Using the Arabidopsis 1001 genomes browser, which contains additional whole genome sequence information, to compare the predicted amino acid sequences of the three *FMO GS-OX* genes between the Col and Sha haplotype groups, amino acid sequence differences between both haplotype groups were observed, which could be responsible for the phenotypic difference (Fig. 4).

## Discussion

The present work was designed to map genomic loci and candidate genes associated with leaf Pi concentration, Pi use efficiency, and leaf sulphate concentrations and to investigate their cross-talk and homeostasis responses to phosphate supply as the environmental variable. Our results show that under -Pi treatment, leaf Pi and sulphate concentrations decrease and both are positively correlated. This is similar to an earlier study that reported a decrease in leaf Pi and sulphate concentrations in Arabidopsis shoots under –Pi treatment [20]. The positive correlation between leaf concentrations of Pi and sulphate can be partly explained by the transcription factor gene *PHR1*. This encodes a MYB-like transcription factor involved in the Pi-response and a central player in the expression regulation of genes involved in Pi transport and remobilization [56]. It also acts as a common regulator of sulphate homeostasis by controlling expression of the sulphate transporter *SULTR* genes in response to Pi starvation [20]. PHR1 contributes to shoot-to-root sulphate transport by up-regulating the expression of *SULTR1;3* and down-regulating the expressions of *SULTR2;1* and *SULTR3;4* [20]. Also in *Brassica napus* the expression of *BnPHR1* is induced by Pi starvation in shoots and roots [57]. Further cross-talk was observed in rice where the disruption of *OsSULTR3;3* reduced phytate and P concentrations and altered the metabolite profile in rice grains [58]. Another SULTR-like transporter, OsSULTR3;4, also named SPDT (SULTR-like P distribution transporter), was reported to be involved in P distribution in rice, and in preferential allocation of P to the grains [59]. In our study, we anticipate that *PHR1*, located on the top of Chr 4, is the causal gene underlying the *PHO2* QTL, especially as it is co-locating with the *SUL5* QTL, with similar effects on leaf Pi and sulphate concentrations. This co-localization would explain pleiotropic effects and provides a possible explanation of the strong coordination between sulphate and Pi signalling pathways under -Pi treatments. Under –Pi treatments, the PHR1 protein is sumoylated by SIZ1, a SUMO E3 ligase protein and a known regulator of P starvation-dependent responses [43]. A significant SNP associated with PUE was also mapped to *SIZ1* in the HapMap/GWAS. Arabidopsis *siz1* mutants show reduced primary root elongation and development of the lateral roots in response to P deprivation, which is evidence of a negative regulatory influence on the auxin distribution pattern [43].

Genes from the *SULTR* family encode the sulphate transporters needed for cellular sulphate uptake. Our HapMap/GWAS results show an association between a SNP mapped to *SULTR2;1* and leaf sulphate concentration. *SULTR2;1* is a major sulphate transporter that co-operates with *SULTR3;5* to transfer sulphate from root to shoot. The effect of this SNP was 5.6 times higher in the -Pi treatment than in the +Pi treatment. Our findings reinforce the proposition that *SULTR2;1* is involved in enhanced sulfolipid biosynthesis and the replacement of phospholipids by sulfolipids under Pi and sulphate deficiency [20, 29], In addition, *SULTR2;1* was reported to be up regulated and overexpressed, mainly in the roots and to lesser extent in the shoots, in response to Pi starvation [20, 60], all supporting its involvement in the response to Pi deficiency.

Intracellular sulphate is needed for glucosinolate biosynthesis [61], requiring the expression of several glucosinolate biosynthesis genes. One of these is the *FLAVIN-MONOOXYGENASE GLUCOSINOLATE S-OXYGENASE* (*FMO GS-OX*) gene family including *FMO GS-OX4* [54, 62]. Our results showed similar allelic effects for the two SNPs mapped to *SULTR2;1* and *FMO GS-OX4* that are associated with leaf sulphate concentration*.* The effects of the non-Col alleles of both SNPs in the -Pi treatment was 5.6 and 7 times higher, respectively, than the effects of the Col alleles in the +Pi treatment, suggesting the anticipation of glucosinolate biosynthesis on sulphate import. The SNP mapped to *FMO GS-OX4* (Fig. 3) is located within the interval of *SUL2*, the major QTL for leaf sulphate concentration mapped to the bottom of Chr 1 (Fig. 1). As the Sha allele for *FMO GS-OX4* / *SUL2* increased the leaf sulphate concentration under –Pi treatment, we expected an amino acid sequence difference between Sha and Col haplotypes for the causal gene and such was indeed found for the *FMO GS-OX4* alleles. Altogether, these results support the candidacy of *FMO GS-OX4* to be the causal gene underlying *SUL2*. However, two paralogues of *FMO GS-OX4, FMO GS-OX2* [55] and *FMO GS-OX3*, with similar functions in sulphate metabolism [54], are flanking this gene, and are also located within the interval of *SUL2*. Since the Sha and Col haplotypes of these *FMO GS-OX2* and *FMO GS-OX3* genes encoding proteins differing in their amino acid sequence, any one, or even all three, *FMO GS-OX* genes could be responsible for the *SUL2* QTL*.*

The same analysis was done for the *5’ADENYLYLPHOSPHOSULFATE REDUCTASE 2* (*APR2*) gene (AT1G62180) [63, 64], which also resides in the *SUL2* confidence interval, but which was not associated with significant SNPs in the HapMap/GWAS. This gene is involved in sulphate reduction [63], and has previously been associated with sulphate content in the Bay x Sha RIL population [63]. Comparing amino acid sequences of *APR2* alleles also revealed differences between the Sha and Col alleles, in line with an earlier report [64] that showed that Sha has a weak allele of *APR2*. However, as this weak allele did not contribute to a difference in leaf sulphate concentration between Sha and Col-0, the authors suggested that further levels of regulation on sulphate accumulation should exist besides *APR2,* which we believe to be one or more of the *FMO GS-OX* genes mapped close by. In addition, the authors also failed to identify any SNPs associated with *APR2* as the causal gene underlying the variation in the concentration of total leaf sulphur using GWAS. Nevertheless, they confirmed the role of *APR2* in sulphate reduction, using bulk segregant analysis combined with SNP microarray genotyping in an F2 population from a cross between the Arabidopsis accessions Col-0 and Hodonin (Hod) [64]. Neither the weak Sha nor the strong Hod hypofunctional *APR2* allele was present in any other accession, indicating these alleles are rare in the global Arabidopsis HapMap/GWAS population and their frequency is too low to be picked up in a GWAS. This also explains why we could not map a significant SNP associated with leaf sulphate concentration to the *APR2* gene.

Another SNP associated with leaf sulphate concentration was mapped to the *Relative of Early Flowering 6* (*REF6*) gene, a positive regulator of flowering in the FLC-dependent pathway [53]. In addition to several reports on pleiotropic effects of *FLC* [65–67] on other traits than flowering time*,* a recent study [68] suggested *FLC*, as well as *SULTR2;1*, as potential candidates underlying a major QTL regulating glucosinolate variation across the life-cycle of *Aethionema arabicum*. In addition to its role in response to sulphur starvation [44, 45], *SULTR2;1* is known to have pleiotropic effects on nitrogen starvation [69] as well as drought and salinity stresses [70]. Pleiotropic effects were also reported for *FMO GS-OX4,* which next to glucosinolate biosynthesis [54, 62] is involved in metal homeostasis [71], and in plant tolerance to freezing [72, 73], salt [74], and drought [75]. We therefore propose pleiotropic effects to be the reason of the co-location of the *PHO1* and *NIT2* QTL, all mapped under –Pi treatment to the top of Chr 2 and the colocation of *PHO3* and *NIT5,* on the top of Chr 5. These pleiotropic effects indicate possible cross-talk between nitrate and Pi and are well supported by recent findings in rice that nitrate-triggered degradation of the Pi signalling repressor SPX4 activates both Pi- and nitrate-responsive genes [76]. Pleiotropic effect may also be the reason for co-localization of *PHO2* with *SUL5* on Chr 4. In both cases, the Col allele was increasing the trait values. This QTL co-locates with the previously mapped PO3.4 QTL, associated with Pi content under different nitrogen availability in the Bay-0 x Sha RIL population [77]. Similarly, *PHO3* and *NIT5* co-locate with NO.10.8, a QTL for nitrogen content, mapped to the top of Chr 5 in the same population [77]. These co-localizations suggest that there may be similar loci involved in controlling the responses to Pi and nitrogen deficiencies. An earlier study [78] reported that both nitrogen and P deficiencies induce accumulation of flavonols in seedling tissue of both *A. thaliana* and tomato. Another study, in sorghum, showed that not only P deficiency, but also nitrogen deficiency, enhanced strigolactone exudation from the root [79]. Several of such co-localizations were found also in the data presented here. For example, three QTLs, *PHO3*, *PUE3* and *NIT5*, co-locate with NO.10.8 [77]. Furthermore, the *SUL1* QTL co-locates with NO.10.1 on the top of Chr 1, and the *SUL2* QTL co-located with PO3.2 on the bottom of Chr 1, while the *SUL4* QTL, mapped on the top of Chr 3, co-located with NO10.6 [77], and with the two QTLs mapped for Pi and phytate [19]. Finally, a QTL for sulphate concentration under normal nitrate treatment mapped to the top of Chr 3 [80], co-located with *CIT1* and *SUL3*.

Next to all the identified co-locating loci there are also several specific loci, indicating that next to common factors in the regulation of Pi deficiency response with nitrogen or sulphur homeostasis, there are also several specific loci, indicating that there are different levels of (co-)regulation, but also illustrating the genetic complexity and polygenic nature of the traits.

## Conclusion

The associations presented here between the studied traits and several genes with known functions related to anion cross-talks and homeostasis in response to Pi availability confirms the suitability of the followed HapMap/GWAS approach to identify candidate genes without the need for additional fine-mapping, as will be needed to resolve the QTLs identified in the RIL population. The SNP mapped to *FMO GS-OX4* is located within the interval of the major QTL for leaf sulphate concentration, *SUL2*, mapped to the bottom of Chr 1. Comparing the amino acid sequences of the *FMO GS-OX2, 3,* and *4* genes, arranged in tandem at this locus, distinguished Col from Sha haplotype groups, supporting the candidacy of at least one of the *FMO GS-OX* genes to be causal for the *SUL2* QTL. Comparing the QTL co-locations observed in the RIL population tested here with earlier studies, indicated possible pleiotropic effects for the QTLs controlling leaf Pi concentration, nitrogen and sulphate concentrations. If such can indeed be confirmed in crops, it would mean breeding for these traits would not need to be done separately, but could be achieved in a few rounds of selection. However, to do so, confirmation of the actual co-localization of QTLs in *A. thaliana*, supported with molecular genetic data, will be needed.

## Methods

### Plant materials and experimental set-up

The Arabidopsis Sha x Col core Recombinant Inbred Line (RIL) population comprising 164 F6 RILs [81] and a diversity panel consisting of 360 world-wide accessions, called the HapMap association panel [82], were used for genetic analysis. The Sha x Col RIL population has been obtained from the Versailles Arabidopsis Stock Center (publiclines.versailles.inra.fr/rils/index), the HapMap set of accessions has been obtained from the European Arabidopsis Stock Centre (arabidopsis.info). The experiments were performed in a completely randomized block design with two replicate blocks for the QTL/RIL population and three replicate blocks for the HapMap/GWAS population. All experiments were conducted in growth chambers set at 12 h day length, a temperature of 20 °C, 60% humidity, and a light intensity of 200 μmoles m^− 2^ s^− 1^. Seeds were stratified for 7 days at 4 °C before being planted on rock wool blocks. The plants were grown under Pi sufficient (+Pi) and deficient (−Pi) treatments. Plants were watered three times per week (at days 2, 4 and 7) for 5 min with a Hyponex nutrient solution (NH_4_^+^, 1.4; K^+^,5.7; Na^+^, 0.2; NO_3_^−^, 5.7 mM; hyponex.co.jp), either supplemented with 1.2 mM KH_2_PO_4_, for +Pi, or with 100 μM KH_2_PO_4_, for –Pi treatments.

### Anion measurements

Plants were grown for 4 weeks, after which their rosettes (shoots) were collected, freeze dried for 48 h in liquid nitrogen and total rosette dry weight (DW) was determined. Six to nine milligrams of the dry material were ground in microfuge tubes and boiled for 15 min at 100 °C with 1 ml of 0.5 N HCl containing 50 mg/l t-aconitate. Samples were centrifuged at 14,000 rpm for 5 min and the supernatant was transferred into 300-μl glass vials. Leaf Pi, IP6, citrate, nitrate, oxalate, and sulphate concentrations were measured using a High-Performance Anion-Exchange Chromatography (HPAE) (Dionex® AS50). Deionized autoclaved water and a 50 mg/l t-aconitate solution were used as a negative control and an internal standard, respectively. Pi Use Efficiency (PUE) was determined as shoot dry weight/leaf Pi concentration [24].

### Statistical analysis and genomic mapping

Statistical analysis was performed using SPSS v21. For each trait, the significant difference between treatments and lines and the significance level for the G × E was tested using analysis of variance [83]. Broad-sense heritability for each trait was estimated as the ratio between the genetic variance *Vg*, and the total phenotypic variance *Vt*, with *Vt* = *Vg* + *Ve*, where *Ve* is the environmental variation, i.e. the variance between replications of each line. A general multi-environment mixed model approach is used for linkage and GWA mapping as previously reported [67, 84]. A single-trait multi-environment approach was followed for QTL mapping in the RIL population, using GenStat for Windows 16th edition (VSN International, Hemel Hempstead, UK) with a –log_10_(*P*) threshold = 2.9, calculated based on the approach implemented in GenStat [85], with 0.05 set as the genome-wide type I error level. However, we report QTLs with lower threshold, i.e. with a minimum of 2.3, if they co-locate with significant QTLs for closely related traits. For GWA mapping, the multi-trait mixed model approach [84] was used, with an arbitrary –log_10_(*P*) threshold of value of 4 and a minor allele frequency of 0.05, which is similar to earlier studies [67, 86, 87], but below the very stringent –log_10_(*P*) = 6.6 threshold when applying a Bonferroni correction for multiple testing assuming independence between SNPs. Genes found to be in LD (LD > 0.3), or within 10 kb on either side of the significant SNP if no LD is found, are listed. A description of all candidate genes was obtained from The Arabidopsis Information Resource (TAIR; www.arabidopsis.org). The Arabidopsis 1001 genomes browser (signal.salk.edu/atg1001/3.0/gebrowser.php) [88] was used to compare the predicted amino acid sequences of all genes co-located with the significant SNPs.

## Additional files

Additional file 1: Population performances, correlations and QTLs for leaf phytate, citrate, nitrate and oxalate concentrations. Overview of comparable results as provided in Tables 1 to 3, for additional anion concentrations. (XLSX 23 kb)

Additional file 2: Frequency distributions of the non-normalized values of the traits measured for the RIL/QTL (a) and HapMap/GWAS (b) populations. Sha-C and Col-C refer to the values of the parental lines grown with sufficient Pi and Sha-D and Col-D to the values of the parental lines grown with deficient Pi, all indicated with arrows. Data for the sufficient Pi treatment are presented with dark grey bars and data from the deficient Pi treatment with light grey bars. The vertical axes indicate the numbers of genotypes per trait value class and the horizontal axes indicate the different trait value classes. IP6 = leaf phytate concentration, CIT = leaf citrate concentration, NIT = leaf nitrate concentration, OXA = leaf oxalate concentration. (PNG 267 kb)

Additional file 3: HapMap/GWA mapping using the multi-trait mixed model approach showing the −log_10_(*P*) values (Y-axis), for all SNPs (X-axis) The measured traits are phytate (a), citrate (b), nitrate (c) and oxalate (d). SNPs associated with candidate genes listed in Additional file 4 are indicated with vertical black lines. In each panel, the SNPs corresponding to the five Arabidopsis chromosomes are indicated in alternating blue/purple colours, with the horizontal axes indicating genome sequence positions. The −log_10_(*P*) arbitrary significance threshold of 4 is indicated with a horizontal dashed line. (PNG 1619 kb)

Additional file 4: List of all candidate genes mapped in the HapMap/GWAS population for anion concentration under phosphate sufficient (+Pi) and deficient (−Pi) treatments. PHO = leaf Pi concentration, PUE = Pi use efficiency, IP6 = leaf phytate concentration, Cit = leaf citrate concentration, Nit = leaf nitrate concentration, Oxa = leaf oxalate concentration, SUL = leaf sulphate concentration. MAF is the minor allele frequency. -log_10_(P) indicates the significance level of association. β indicates the phenotypic effect of a SNP in (+Pi) or (−Pi) with positive values indicating a positive effect on the trait value from the Col allele. Chromosome numbers are indicated (Chr.). The SNP positions on each chromosome are given, in kilo basepairs (kb). Both SNP position and description is based on TAIR v.10 (www.arabidopsis.org). Genes found to be in linkage disequilibrium (LD), (LD > 0.3) or within 10 kb on both sides of the significant SNP if no LD is found, are listed. (XLSX 38 kb)

## Data Availability

The datasets used and/or analysed during the current study are available from the corresponding author on reasonable request.

## Abbreviations

+Pi/−Pi: Sufficient/deficient phosphate supply
DW: Dry weight
GWAS: Genome wide association studies
GxE: Genotype by environment interaction
IP6: Phytate
Kb: Kilobasepair
LD: Linkage disequilibrium
P: Phosphorous
PHO: Leaf Pi concentration
Pi: Inorganic phosphate
PUE: Phosphate use efficiency
QTL: Quantitative trait locus
RIL: Recombinant inbred line
SNP: Single nucleotide polymorphism
SUL: Leaf sulphate concentration
TAIR: The Arabidopsis Information Resource (www.arabidopsis.org)

## References

[1] Baligar VC, Fageria NK, He ZL (2001). Nutrient use efficiency in plants. Commun Soil Sci Plant Anal.

[2] Heuer S, Gaxiola R, Schilling R, Herrera-Estrella L, López-Arredondo D, Wissuwa M, Delhaize E, Rouached H (2017). Improving phosphorus use efficiency: a complex trait with emerging opportunities. Plant J.

[3] Gerke J (2015). The acquisition of phosphate by higher plants: effect of carboxylate release by the roots. A critical review. J Plant Nutr Soil Sci.

[4] Weihrauch C, Opp C (2018). Ecologically relevant phosphorus pools in soils and their dynamics: the story so far. Geoderma.

[5] Uhde-Stone C, Zinn KE, Ramirez-Yáñez M, Li A, Vance CP, Allan DL (2003). Nylon filter arrays reveal differential gene expression in proteoid roots of white lupin in response to phosphorus deficiency. Plant Physiol.

[6] Hammond JP, White PJ (2008). Sucrose transport in the phloem: integrating root responses to phosphorus starvation. J Exp Bot.

[7] Singh Gahoonia T, Nielsen NE (2004). Root traits as tools for creating phosphorus efficient crop varieties. Plant Soil.

[8] Secco David, Bouain Nadia, Rouached Aida, Prom-u-thai Chanakan, Hanin Moez, Pandey Ajay K., Rouached Hatem (2017). Phosphate, phytate and phytases in plants: from fundamental knowledge gained in Arabidopsis to potential biotechnological applications in wheat. Critical Reviews in Biotechnology.

[9] Gilbert N (2009). Environment: the disappearing nutrient. Nature.

[10] Alatorre-Cobos F, López-Arredondo D, Herrera-Estrella L. Genetic determinants of phosphate use efficiency in crops. In: Genes for Plant Abiotic Stress. Hoboken: Wiley-Blackwell; 2009. p. 143–65.

[11] Margenot AJ, Sommer R, Mukalama J, Parikh SJ (2017). Biological P cycling is influenced by the form of P fertilizer in an Oxisol. Biol Fertil Soils.

[12] Sanyal SK, De Datta SK, Stewart BA (1991). Chemistry of Phosphorus Transformations in Soil. Advances in Soil Science.

[13] Ryan PR, Tyerman SD, Sasaki T, Furuichi T, Yamamoto Y, Zhang WH, Delhaize E (2011). The identification of aluminium-resistance genes provides opportunities for enhancing crop production on acid soils. J Exp Bot.

[14] Jemo M, Abaidoo RC, Nolte C, Horst WJ (2007). Aluminum resistance of cowpea as affected by phosphorus-deficiency stress. J Plant Physiol.

[15] Neumann G, Massonneau A, Martinoia E, Römheld V (1999). Physiological adaptations to phosphorus deficiency during proteoid root development in white lupin. Planta.

[16] Kirkby EA, Johnston AE, White PJ, Hammond JP (2008). Soil and fertilizer phosphorus in relation to crop nutrition. The Ecophysiology of Plant-Phosphorus Interactions.

[17] Liao H, Wan H, Shaff J, Wang X, Yan X, Kochian LV (2006). Phosphorus and aluminum interactions in soybean in relation to aluminum tolerance. Exudation of specific organic acids from different regions of the intact root system. Plant Physiol.

[18] Lin ZH, Chen LS, Chen RB, Zhang FZ, Jiang HX, Tang N, Smith BR (2011). Root release and metabolism of organic acids in tea plants in response to phosphorus supply. J Plant Physiol.

[19] Bentsink L, Yuan K, Koornneef M, Vreugdenhil D (2003). The genetics of phytate and phosphate accumulation in seeds and leaves of Arabidopsis thaliana, using natural variation. Theor Appl Genet.

[20] Rouached H, Secco D, Arpat B, Poirier Y (2011). The transcription factor PHR1 plays a key role in the regulation of sulfate shoot-to-root flux upon phosphate starvation in Arabidopsis. BMC Plant Biol.

[21] Poza-Carrión C, Paz-Ares J (2019). When nitrate and phosphate sensors meet. Nat Plants.

[22] Gomez-Roldan V, Fermas S, Brewer PB, Puech-Pages V, Dun EA, Pillot JP, Letisse F, Matusova R, Danoun S, Portais JC (2008). Strigolactone inhibition of shoot branching. Nature.

[23] Ramaekers L, Remans R, Rao IM, Blair MW, Vanderleyden J (2010). Strategies for improving phosphorus acquisition efficiency of crop plants. Field Crop Res.

[24] Gourley CJP, Allan DL, Russelle MP (1994). Plant nutrient efficiency: a comparison of definitions and suggested improvement. Plant Soil.

[25] Uzokwe VNE, Asafo-Adjei B, Fawole I, Abaidoo R, Odeh IOA, Ojo DK, Dashiell K, Sanginga N (2017). Generation mean analysis of phosphorus-use efficiency in freely nodulating soybean crosses grown in low-phosphorus soil. Plant Breed.

[26] Wang F, Deng M, Xu J, Zhu X, Mao C (2018). Molecular mechanisms of phosphate transport and signaling in higher plants. Semin Cell Dev Biol.

[27] Raghothama KG (1999). Phosphate acquisition. Annu Rev Plant Phys.

[28] Puga MI, Rojas-Triana M, de Lorenzo L, Leyva A, Rubio V, Paz-Ares J (2017). Novel signals in the regulation of pi starvation responses in plants: facts and promises. Curr Opin Plant Biol.

[29] Briat JF, Rouached H, Tissot N, Gaymard F, Dubos C (2015). Integration of P, S, Fe, and Zn nutrition signals in Arabidopsis thaliana: potential involvement of PHOSPHATE STARVATION RESPONSE 1 (PHR1). Front Plant Sci.

[30] El-Soda M, Malosetti M, Zwaan BJ, Koornneef M, Aarts MG (2014). Genotype x environment interaction QTL mapping in plants: lessons from Arabidopsis. Trends Plant Sci.

[31] Hammond JP, Bennett MJ, Bowen HC, Broadley MR, Eastwood DC, May ST, Rahn C, Swarup R, Woolaway KE, White PJ (2003). Changes in gene expression in Arabidopsis shoots during phosphate starvation and the potential for developing smart plants. Plant Physiol.

[32] Bonnot C, Pinson B, Clément M, Bernillon S, Chiarenza S, Kanno S, Kobayashi N, Delannoy E, Nakanishi TM, Nussaume L (2016). A chemical genetic strategy identify the PHOSTIN, a synthetic molecule that triggers phosphate starvation responses in Arabidopsis thaliana. New Phytol.

[33] Ayadi A, David P, Arrighi J-F, Chiarenza S, Thibaud M-C, Nussaume L, Marin E (2015). Reducing the genetic redundancy of Arabidopsis PHOSPHATE TRANSPORTER1 transporters to study phosphate uptake and signaling. Plant Physiol.

[34] Sun L, Song L, Zhang Y, Zheng Z, Liu D (2016). Arabidopsis PHL2 and PHR1 act redundantly as the key components of the central regulatory system controlling transcriptional responses to phosphate starvation. Plant Physiol.

[35] Hammond JP, Mayes S, Bowen HC, Graham NS, Hayden RM, Love CG, Spracklen WP, Wang J, Welham SJ, White PJ (2011). Regulatory hotspots are associated with plant gene expression under varying soil phosphorus supply in Brassica rapa. Plant Physiol.

[36] Ning L, Kan G, Du W, Guo S, Wang Q, Zhang G, Cheng H, Yu D (2016). Association analysis for detecting significant single nucleotide polymorphisms for phosphorus-deficiency tolerance at the seedling stage in soybean [Glycine max (L) Merr.]. Breed Sci.

[37] Eichler EE, Flint J, Gibson G, Kong A, Leal SM, Moore JH, Nadeau JH (2010). Missing heritability and strategies for finding the underlying causes of complex disease. Nat Rev Genet.

[38] Gibson G (2012). Rare and common variants: twenty arguments. Nat Rev Genet.

[39] Brachi B, Faure N, Horton M, Flahauw E, Vazquez A, Nordborg M, Bergelson J, Cuguen J, Roux F (2010). Linkage and association mapping of *Arabidopsis thaliana* flowering time in nature. PLoS Genet.

[40] Arino J, Perez-Callejon E, Cunillera N, Camps M, Posas F, Ferrer A (1993). Protein phosphatases in higher plants: multiplicity of type 2A phosphatases in Arabidopsis thaliana. Plant Mol Biol.

[41] Sottosanto JB, Gelli A, Blumwald E (2004). DNA array analyses of Arabidopsis thaliana lacking a vacuolar Na+/H+ antiporter: impact of AtNHX1 on gene expression. Plant J.

[42] Liu L, Yan X, Kong X, Zhao Y, Gong Z, Jin JB, Guo Y (2017). Transcriptional gene silencing maintained by OTS1 SUMO protease requires a DNA-dependent polymerase V-dependent pathway. Plant Physiol.

[43] Miura K, Rus A, Sharkhuu A, Yokoi S, Karthikeyan AS, Raghothama KG, Baek D, Koo YD, Jin JB, Bressan RA (2005). The Arabidopsis SUMO E3 ligase SIZ1 controls phosphate deficiency responses. Proc Natl Acad Sci U S A.

[44] Maruyama-Nakashita A, Watanabe-Takahashi A, Inoue E, Yamaya T, Saito K, Takahashi H (2015). Sulfur-responsive elements in the 3′-nontranscribed intergenic region are essential for the induction of SULFATE TRANSPORTER 2;1 gene expression in Arabidopsis roots under sulfur deficiency. Plant Cell.

[45] Kawashima CG, Yoshimoto N, Maruyama-Nakashita A, Tsuchiya YN, Saito K, Takahashi H, Dalmay T (2009). Sulphur starvation induces the expression of microRNA-395 and one of its target genes but in different cell types. Plant J.

[46] Silver DM, Kotting O, Moorhead GB (2014). Phosphoglucan phosphatase function sheds light on starch degradation. Trends Plant Sci.

[47] Silver DM, Silva LP, Issakidis-Bourguet E, Glaring MA, Schriemer DC, Moorhead GBG (2013). Insight into the redox regulation of the phosphoglucan phosphatase SEX4 involved in starch degradation. FEBS J.

[48] Santelia D, Kötting O, Seung D, Schubert M, Thalmann M, Bischof S, Meekins DA, Lutz A, Patron N, Gentry MS (2011). The phosphoglucan phosphatase like sex four2 dephosphorylates starch at the C3-position in Arabidopsis. Plant Cell.

[49] Kerk D, Conley TR, Rodriguez FA, Tran HT, Nimick M, Muench DG, Moorhead GBG (2006). A chloroplast-localized dual-specificity protein phosphatase in Arabidopsis contains a phylogenetically dispersed and ancient carbohydrate-binding domain, which binds the polysaccharide starch. Plant J.

[50] Ichimura K, Shinozaki K, Tena G, Sheen J, Henry Y, Champion A, Kreis M, Zhang S, Hirt H, Wilson C (2002). Mitogen-activated protein kinase cascades in plants: a new nomenclature. Trends Plant Sci.

[51] Nemoto K, Seto T, Takahashi H, Nozawa A, Seki M, Shinozaki K, Endo Y, Sawasaki T (2011). Autophosphorylation profiling of Arabidopsis protein kinases using the cell-free system. Phytochemistry.

[52] Lu F, Cui X, Zhang S, Jenuwein T, Cao X (2011). Arabidopsis REF6 is a histone H3 lysine 27 demethylase. Nat Genet.

[53] Noh B, Lee S-H, Kim H-J, Yi G, Shin E-A, Lee M, Jung K-J, Doyle MR, Amasino RM, Noh Y-S (2004). Divergent roles of a pair of homologous jumonji/zinc-finger–class transcription factor proteins in the regulation of Arabidopsis flowering time. Plant Cell.

[54] Li J, Hansen BG, Ober JA, Kliebenstein DJ, Halkier BA (2008). Subclade of flavin-monooxygenases involved in aliphatic glucosinolate biosynthesis. Plant Physiol.

[55] Hansen BG, Kliebenstein DJ, Halkier BA (2007). Identification of a flavin-monooxygenase as the S-oxygenating enzyme in aliphatic glucosinolate biosynthesis in Arabidopsis. Plant J.

[56] Rubio V, Linhares F, Solano R, Martín AC, Iglesias J, Leyva A, Paz-Ares J (2001). A conserved MYB transcription factor involved in phosphate starvation signaling both in vascular plants and in unicellular algae. Genes Dev.

[57] Ren F, Guo Q-Q, Chang L-L, Chen L, Zhao C-Z, Zhong H, Li X-B (2012). Brassica napus PHR1 gene encoding a MYB-like protein functions in response to phosphate starvation. PLoS One.

[58] Zhao H, Frank T, Tan Y, Zhou C, Jabnoune M, Arpat AB, Cui H, Huang J, He Z, Poirier Y (2016). Disruption of OsSULTR3;3 reduces phytate and phosphorus concentrations and alters the metabolite profile in rice grains. New Phytol.

[59] Yamaji N, Takemoto Y, Miyaji T, Mitani-Ueno N, Yoshida KT, Ma JF (2017). Reducing phosphorus accumulation in rice grains with an impaired transporter in the node. Nature.

[60] Hsieh LC, Lin SI, Shih AC, Chen JW, Lin WY, Tseng CY, Li WH, Chiou TJ (2009). Uncovering small RNA-mediated responses to phosphate deficiency in Arabidopsis by deep sequencing. Plant Physiol.

[61] Davidian J-C, Kopriva S (2010). Regulation of sulfate uptake and assimilation—the same or not the same?. Mol Plant.

[62] Li J, Kristiansen KA, Hansen BG, Halkier BA (2011). Cellular and subcellular localization of flavin-monooxygenases involved in glucosinolate biosynthesis. J Exp Bot.

[63] Loudet O, Saliba-Colombani V, Camilleri C, Calenge F, Gaudon V, Koprivova A, North KA, Kopriva S, Daniel-Vedele F (2007). Natural variation for sulfate content in Arabidopsis thaliana is highly controlled by APR2. Nat Genet.

[64] Chao D-Y, Baraniecka P, Danku J, Koprivova A, Lahner B, Luo H, Yakubova E, Dilkes B, Kopriva S, Salt DE (2014). Variation in sulfur and selenium accumulation is controlled by naturally occurring isoforms of the key sulfur assimilation enzyme ADENOSINE 5′-PHOSPHOSULFATE REDUCTASE2 across the Arabidopsis species range. Plant Physiol.

[65] Boss PK, Bastow RM, Mylne JS, Dean C (2004). Multiple pathways in the decision to flower: enabling, promoting, and resetting. Plant Cell.

[66] Salehi H, Ransom CB, Oraby HF, Seddighi Z, Sticklen MB (2005). Delay in flowering and increase in biomass of transgenic tobacco expressing the Arabidopsis floral repressor gene FLOWERING LOCUS C. J Plant Physiol.

[67] El-Soda M, Willem K, Malosetti M, Koornneef M, Aarts MGM (2015). Quantitative trait loci and candidate genes underlying genotype by environment interaction in the response of Arabidopsis thaliana to drought. Plant Cell Environ.

[68] Mohammadin S, Nguyen T-P, van Weij MS, Reichelt M, Schranz ME. Flowering Locus C (FLC) Is a Potential Major Regulator of Glucosinolate Content across Developmental Stages of Aethionema arabicum (Brassicaceae). Front Plant Sci. 2017;8:876. 10.3389/fpls.2017.00876 PMC5445170 28603537

[69] Peng M, Bi Y-M, Zhu T, Rothstein S (2007). Genome-wide analysis of Arabidopsis responsive transcriptome to nitrogen limitation and its regulation by the ubiquitin ligase gene NLA. Plant Mol Biol.

[70] Gallardo K, Courty P-E, Le Signor C, Wipf D, Vernoud V. Sulfate transporters in the plant’s response to drought and salinity: regulation and possible functions. Front Plant Sci. 2014;5:580. 10.3389/fpls.2014.00580 PMC4212607 25400648

[71] Becher M, Talke IN, Krall L, Kramer U (2004). Cross-species microarray transcript profiling reveals high constitutive expression of metal homeostasis genes in shoots of the zinc hyperaccumulator Arabidopsis halleri. Plant J.

[72] Oono Y, Seki M, Satou M, Iida K, Akiyama K, Sakurai T, Fujita M, Yamaguchi-Shinozaki K, Shinozaki K (2006). Monitoring expression profiles of Arabidopsis genes during cold acclimation and deacclimation using DNA microarrays. Funct Integr Genomics.

[73] Zhang X, Fowler SG, Cheng H, Lou Y, Rhee SY, Stockinger EJ, Thomashow MF (2004). Freezing-sensitive tomato has a functional CBF cold response pathway, but a CBF regulon that differs from that of freezing-tolerant Arabidopsis. Plant J.

[74] Taji T, Seki M, Satou M, Sakurai T, Kobayashi M, Ishiyama K, Narusaka Y, Narusaka M, Zhu J-K, Shinozaki K (2004). Comparative genomics in Salt tolerance between Arabidopsis and Arabidopsis-related halophyte Salt cress using Arabidopsis microarray. Plant Physiol.

[75] Huang D, Wu W, Abrams SR, Cutler AJ (2008). The relationship of drought-related gene expression in Arabidopsis thaliana to hormonal and environmental factors. J Exp Bot.

[76] Hu B, Jiang Z, Wang W, Qiu Y, Zhang Z, Liu Y, Li A, Gao X, Liu L, Qian Y (2019). Nitrate-NRT1.1B-SPX4 cascade integrates nitrogen and phosphorus signalling networks in plants. Nat Plants.

[77] Loudet O, Chaillou S, Krapp A, Daniel-Vedele F (2003). Quantitative trait loci analysis of water and anion contents in interaction with nitrogen availability in *Arabidopsis thaliana*. Genetics.

[78] Stewart AJ, Chapman W, Jenkins GI, Graham I, Martin T, Crozier A (2001). The effect of nitrogen and phosphorus deficiency on flavonol accumulation in plant tissues. Plant Cell Environ.

[79] Yoneyama K, Xie X, Kusumoto D, Sekimoto H, Sugimoto Y, Takeuchi Y, Yoneyama K (2007). Nitrogen deficiency as well as phosphorus deficiency in sorghum promotes the production and exudation of 5-deoxystrigol, the host recognition signal for arbuscular mycorrhizal fungi and root parasites. Planta.

[80] Koprivova A, Giovannetti M, Baraniecka P, Lee BR, Grondin C, Loudet O, Kopriva S (2013). Natural variation in the ATPS1 isoform of ATP sulfurylase contributes to the control of sulfate levels in Arabidopsis. Plant Physiol.

[81] Simon M, Loudet O, Durand S, Bérard A, Brunel D, Sennesal F-X, Durand-Tardif M, Pelletier G, Camilleri C (2008). Quantitative trait loci mapping in five new large recombinant inbred line populations of Arabidopsis thaliana genotyped with consensus single-nucleotide polymorphism markers. Genetics.

[82] Li Y, Huang Y, Bergelson J, Nordborg M, Borevitz JO (2010). Association mapping of local climate-sensitive quantitative trait loci in *Arabidopsis thaliana*. Proc Natl Acad Sci.

[83] Giraud E, Ho LHM, Clifton R, Carroll A, Estavillo G, Tan Y-F, Howell KA, Ivanova A, Pogson BJ, Millar AH (2008). The absence of ALTERNATIVE OXIDASE1a in Arabidopsis results in acute sensitivity to combined light and drought stress. Plant Physiol.

[84] Korte A, Vilhjalmsson BJ, Segura V, Platt A, Long Q, Nordborg M (2012). A mixed-model approach for genome-wide association studies of correlated traits in structured populations. Nat Genet.

[85] Li J, Ji L (2005). Adjusting multiple testing in multilocus analyses using the eigenvalues of a correlation matrix. Heredity.

[86] Olivas NHD, Kruijer W, Gort G, Wijnen CL, Loon JJA, Dicke M (2017). Genome-wide association analysis reveals distinct genetic architectures for single and combined stress responses in Arabidopsis thaliana. New Phytol.

[87] Kooke R, Kruijer W, Bours R, Becker F, Kuhn A, van de Geest H, Buntjer J, Doeswijk T, Guerra J, Bouwmeester H (2016). Genome-wide association mapping and genomic prediction elucidate the genetic architecture of morphological traits in Arabidopsis. Plant Physiol.

[88] Weigel D, Mott R (2009). The 1001 genomes project for *Arabidopsis thaliana*. Genome Biol.

