# Supplementary figures and images for: QTL and candidate genes associated with leaf anion concentrations in response to phosphate supply in Arabidopsis thaliana
Source: BMC Plant Biol. 2019 Sep 18;19:410. doi: 10.1186/s12870-019-1996-3 (PMC6751748; doi:10.1186/s12870-019-1996-3)

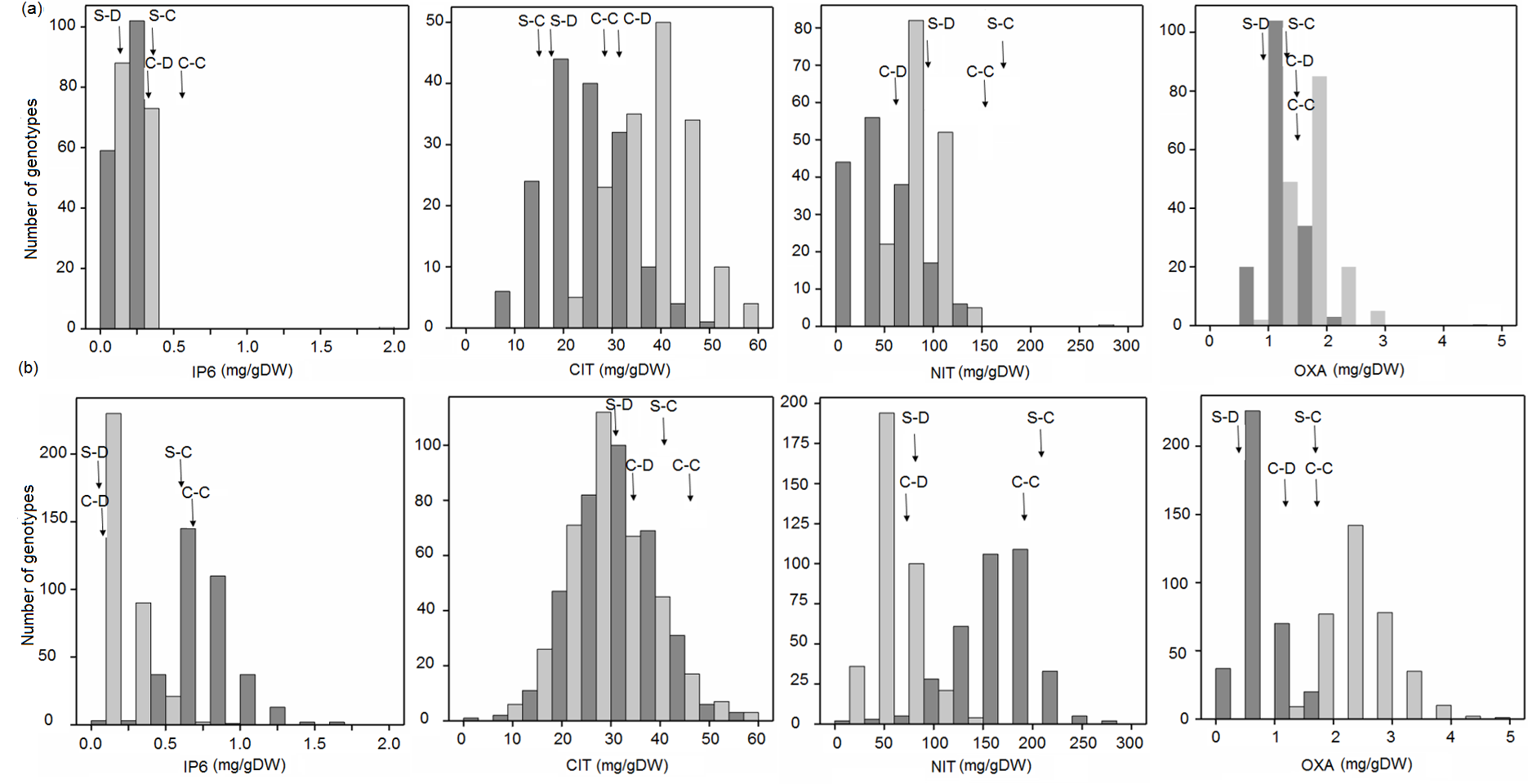

Supplement: Supplementary file 2 — Frequency distributions of the non-normalized values of the traits measured for the RIL/QTL (a) and HapMap/GWAS (b) populations. Sha-C and Col-C refer to the values of the parental lines grown with sufficient Pi and Sha-D and Col-D to the values of the parental lines grown with deficient Pi, all indicated with arrows. Data for the sufficient Pi treatment are presented with dark grey bars and data from the deficient Pi treatment with light grey bars. The vertical axes indicate the numbers of genotypes per trait value class and the horizontal axes indicate the different trait value classes. IP6 = leaf phytate concentration, CIT = leaf citrate concentration, NIT = leaf nitrate concentration, OXA = leaf oxalate concentration. (PNG 267 kb) [file 12870_2019_1996_MOESM2_ESM.png]

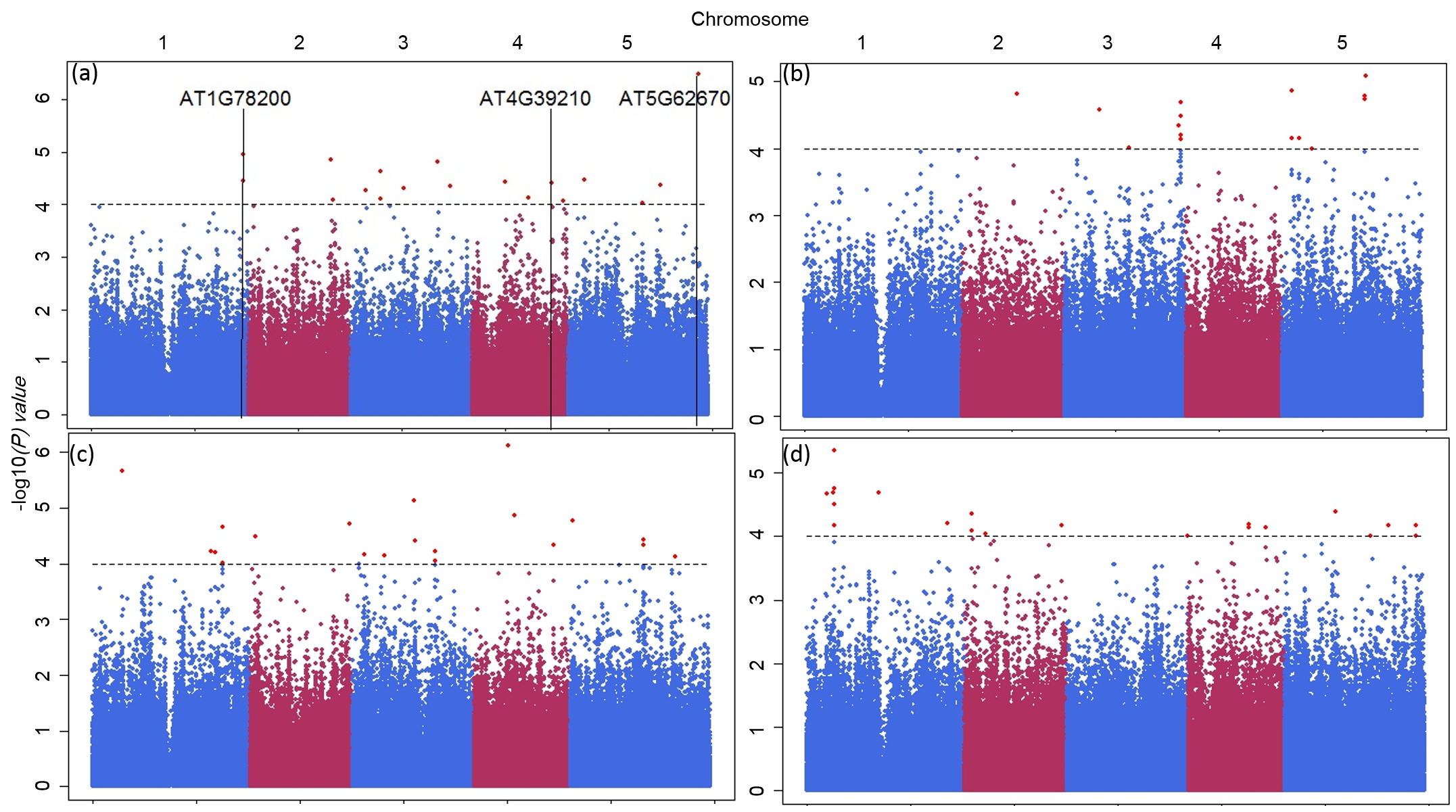

Supplement: Supplementary file 3 — HapMap/GWA mapping using the multi-trait mixed model approach showing the −log10(P) values (Y-axis), for all SNPs (X-axis) The measured traits are phytate (a), citrate (b), nitrate (c) and oxalate (d). SNPs associated with candidate genes listed in Additional file 4 are indicated with vertical black lines. In each panel, the SNPs corresponding to the five Arabidopsis chromosomes are indicated in alternating blue/purple colours, with the horizontal axes indicating genome sequence positions. The −log10(P) arbitrary significance threshold of 4 is indicated with a horizontal dashed line. (PNG 1619 kb) [file 12870_2019_1996_MOESM3_ESM.png]
